# Evaluation of Two Injection Techniques in Combination with the Local Anesthetics Lidocaine and Mepivacaine for Piglets Undergoing Surgical Castration

**DOI:** 10.3390/ani12081028

**Published:** 2022-04-14

**Authors:** Julia Werner, Anna M. Saller, Judith Reiser, Steffanie Senf, Pauline Deffner, Nora Abendschön, Johannes Fischer, Andrea Grott, Regina Miller, Yury Zablotski, Katja Steiger, Shana Bergmann, Michael H. Erhard, Mathias Ritzmann, Susanne Zöls, Christine Baumgartner

**Affiliations:** 1 Center of Preclinical Research, Technical University of Munich, 81675 Munich, Germany; julia.werner@tum.de (J.W.); anna.saller@tum.de (A.M.S.); judith.reiser@tum.de (J.R.); fischer.johannes@tum.de (J.F.); 2 Clinic for Swine, Center for Clinical Veterinary Medicine, LMU Munich, 85764 Oberschleissheim, Germany; s.senf@med.vetmed.uni-muenchen.de (S.S.); p.deffner@med.vetmed.uni-muenchen.de (P.D.); nora.abendschoen@gmail.com (N.A.); y.zablotski@med.vetmed.uni-muenchen.de (Y.Z.); ritzmann@med.vetmed.uni-muenchen.de (M.R.); s.zoels@med.vetmed.uni-muenchen.de (S.Z.); 3 Chair of Animal Welfare, Ethology, Animal Hygiene and Husbandry, LMU Munich, 80539 Munich, Germany; a.schoerwerth@tierhyg.vetmed.uni-muenchen.de (A.G.); r.miller@tierhyg.vetmed.uni-muenchen.de (R.M.); s.bergmann@tierhyg.vetmed.uni-muenchen.de (S.B.); m.erhard@tierhyg.vetmed.uni-muenchen.de (M.H.E.); 4 Institute of Pathology, School of Medicine, Technical University of Munich, 81675 Munich, Germany; katja.steiger@tum.de

**Keywords:** piglet, castration, local anesthesia, pain, blood pressure, heart rate, defensive movements, fos protein, distribution, injection

## Abstract

**Simple Summary:**

Pain elimination during the surgical castration of male piglets is an important animal welfare issue. Bans on castration without pain relief are pending in most pork-producing European countries. Surgical castration of suckling piglets without anesthesia has been prohibited in Germany since January 2021. Boar fattening, gonadotropin-releasing hormone (GnRH) vaccination and surgical castration under isoflurane or injection anesthesia are permitted as legal alternatives. These alternatives have advantages and disadvantages, and a feasible and simple method is still needed. The legal basis for another alternative, i.e., the application of a local anesthetic by the farmer prior to castration, has been created in Germany, but evidence of its efficacy remains controversial. The present study developed a feasible and effective method for local anesthesia in piglet castration. Two different injection techniques in combination with the local anesthetics lidocaine and mepivacaine were investigated using nociceptive parameters, injection pressure and tissue distribution. Both injection techniques significantly reduced the nociceptive parameters regardless of the local anesthetic used and achieved similar distribution patterns and injection pressures. One method performed slightly better in the experimental setup, based on injection duration and handling.

**Abstract:**

The present study evaluated the effects of two injection techniques in combination with lidocaine or mepivacaine for piglets undergoing castration. To improve ease of use, a cannula with side holes (one-step fenestrated (F)) was invented to simultaneously deliver a local anesthetic into the testis and scrotum and was compared to a two-step injection technique. The distribution of a lidocaine/contrast agent mixture using the two methods was examined using computed tomography. Piglets were randomly divided into treatment groups: handling, castration without pain relief and castration after lidocaine or mepivacaine injection using the one-step F or two-step method. Acute physiological responses to noxious stimuli were evaluated by measuring the mean arterial blood pressure (MAP), heart rate (HR) and nocifensive movements. Fos protein expression in the spinal dorsal cord was semi-quantitatively analyzed. Both injection techniques achieved similar distribution patterns. The one-step F method was faster and easier. Injection was not associated with significant changes in MAP or HR, but Mepi1 and NaCl elicited significantly increased nocifensive movements. Both techniques significantly reduced MAP and nocifensive movements when the spermatic cords were cut, regardless of the local anesthetic type. Compared to NaCl, only the lidocaine treatments significantly reduced HR during skin incision. Lido2 significantly reduced Fos protein expression.

## 1. Introduction

Surgical castration of male piglets without pain relief is a major animal welfare concern. Based on the current knowledge, surgical castration is considered a painful procedure regardless of the piglet’s age [1,2]. The main reasons for the castration of male piglets are to avoid boar taint in carcasses, to reduce aggressive and sexual behaviors that are specific to intact males, and to ensure constant meat quality [3,4,5,6]. European legislation allows surgical piglet castration without anesthetic up to an age of 7 days [7]. There is a growing consensus to completely ban surgical piglet castration by implementing the practice of boar fattening or immunocastration. However, surgical castration remains a common husbandry procedure in Europe, but it is handled very differently in individual member states. Bans on castration without pain relief are still pending in most pork-producing European countries [8]. Several European countries have recently committed to abandoning the practice of surgical castration without anesthesia. Surgical castration does not play a major role in Portugal, Spain, Ireland, the United Kingdom and the Netherlands because the production of boars is common [8,9].

Common anesthetic approaches for surgical piglet castration are inhalational anesthesia with isoflurane (Switzerland, Germany) or CO_2_/O_2_ (Netherlands) or injection anesthesia (Croatia, Bulgaria, Germany, Portugal, Switzerland) [9]. Another alternative is the application of a local anesthetic prior to castration, which is practiced in some Scandinavian countries [8,10]. The legal requirements for the use of local anesthesia for piglet castration are stricter in Germany because they necessitate not only pain relief but effective pain elimination during castration. Local anesthesia is not permitted for piglet castration in Germany for the following reasons: evidence of efficacy is controversial, and no approved veterinary local anesthetic product for piglet castration is currently available on the German market. Several studies have investigated local anesthesia for piglet castration and reported divergent results [10,11,12,13,14,15,16,17,18]. A meaningful comparison of the data is difficult due to different study protocols, such as the choice of local anesthetics, injection techniques and outcome measures. Further studies and approaches are needed to confirm effective pain elimination during castration.

The present study is based on the results of two previous studies, which evaluated the effects of four local anesthetics in isoflurane-anesthetized and awake piglets undergoing castration [19,20]. Only lidocaine and mepivacaine were further investigated in the present study because these agents showed the most promising results for pain reduction during castration. Both local anesthetics achieved significant reductions in mean arterial blood pressure (MAP) during all castration steps under light isoflurane anesthesia and produced a decrease in heart rate (HR) changes during castration, except for lidocaine after skin incision [19]. Both local anesthetics reduced defensive behavior during skin incisions and cutting of the spermatic cord in conscious piglets [20].

However, the injection technique (intratesticular injection of 0.5 mL, retracting cannula into subscrotal tissue, making a skin fold and injection of 0.5 mL subscrotally) used by Saller et al. [19] and Abendschön et al. [20] appeared to be challenging in awake piglets. The injection technique induced nocifensive behavior in response to the injection of a local anesthetic. Therefore, the present study investigated a more practical and less painful method for the injection of local anesthesia while maintaining the efficacy demonstrated in Saller and Abendschön [19,20]. The injection volume was reduced from 1 mL to 0.6 mL per side, and two different injection methods were evaluated. One technique used a two-step injection process modified by Hansson et al. [11]. The other technique used a specially designed cannula to simultaneously apply a local anesthetic intratesticularly and subcutaneously in only one step (the one-step fenestrated, or F, method).

The present study evaluated and compared two different injection techniques and two local anesthetics for effectiveness during piglet castration and improvement of injection pain based on cardiovascular changes, nocifensive movements and Fos expression. Tissue distribution by contrast-enhanced computed tomography (CT) and injection pressure of both application methods were also compared.

## 2. Materials and Methods

### 2.1. Animals

Fifty-two clinically healthy hybrid German Landrace/German Large White x Pietrain male piglets aged 3 to 7 days (4.8 ± 1.3 days) were included in the castration study. Twelve piglets were used for semiquantitative distribution experiments using CT imaging. Sows and piglets were housed in the animal husbandry unit of the Clinic for Swine (Oberschleißheim, Bavaria, Germany), according to the German Order for the Keeping of Productive Animals and the EU Directive 2010/63/EU for animal experiments.

For all piglets, the following additional inclusion criteria were applied: a body weight over 1.4 kg (mean: 2.1 kg ± 0.5 kg) and two testicles without any deviation from the normal anatomical condition. Piglets received an oral bolus of iron (1 mL per piglet; Ursoferran^®^ 150 mg/mL, Serumwerk Bernburg AG, Bernburg, Germany) during the first 10 h of life. No tooth clipping, ear tagging or tail docking was performed. The study was performed in compliance with EU Directive 2010/63/EU for animal experiments and the German Animal Welfare Act (2020). The Ethical Committee for Animal Experiments of the Government of Upper Bavaria, Munich, Germany, approved the experiments (Reference Number ROB-55.2-2532.Vet_02-19-11).

### 2.2. Distribution Study

To investigate the distribution of a local anesthetic applied using the two different injection techniques, 12 isoflurane-anesthetized piglets were randomly assigned to the two injection methods (n = 6). A total volume of 0.6 mL comprised of a mixture of contrast agent (Imeron^®^ 300 M, 300 mg Iod/mL, Injektionslösung Iomeprol, Bracco Imaging Deutschland GmbH, Konstanz, Germany) diluted 1:10 in lidocaine hydrochloride 2% (Xylocitin^®^ 2%, Mibe GmbH Arzneimittel, Sandersdorf-Brehna, Germany) was injected with either the one-step F or two-step method as described below. The same two people performed fixation and injection. Piglets received an intramuscular dose of 50 mg/kg metamizole (METAMIZOL WDT, 500 mg/mL, Injektionslösung für Pferde, Rinder, Schweine und Hunde, WDT—Wirtschaftsgenossenschaft deutscher Tierärzte eG, Hanover, Germany) 30 min prior to the induction of anesthesia. CT was performed using a dual-layer spectral CT (IQon Spectral-CT, Philips Healthcare, Best, The Netherlands) every two minutes until 20 min after injection. The following evaluation parameters were used for all CT scans: 120 kV, 74 mA and 2-mm thickness.

Image analysis was performed using a standard DICOM Viewer (LARA View, DEKOM Medical, Hamburg, Germany), and 3D reconstruction was performed using semiautomated software (IntelliSpace PACS, Philips Healthcare, Best, the Netherlands). Three independent and blinded raters performed semiquantitative analyses using a CT distribution scoring system (Table 1). Directly after injection and after 20 min, CT scans were evaluated for the distribution and accumulation of the contrast agent in the testicular area.

### 2.3. Injection Pressure and Injection Time

The injection pressure of the two methods used in the experiments was measured in four piglets injected using the two-step method and seven piglets injected using the one-step F method. Immediately after euthanasia injection with 0.6 mL 0.9% sodium chloride (B. Braun, Melsungen, Germany) was performed via the one-step F or two-step method, as described. Before injection, the system was flushed with 0.9% sodium chloride, and all air bubbles were removed from the hoses. Pressure was measured via a pressure sensor (Xtrans, Codan pvb Medical GmbH) and recorded every four seconds (EIM-B, EIM-A, HAEMODYN software, Hugo Sachs Elektronik—Harvard Apparatus GmbH, March-Hugstetten, Germany). Injections into the air were performed to determine the baseline system pressure due to the equipment. The mean maximum pressures of both injection techniques into the testicular tissue and air were calculated. To determine the duration of injection, the time from injection into the first testis to withdrawal of the cannula from the second testis was measured.

### 2.4. Castration Study

This was a randomized, double-blind experimental study. A computer program for simple randomization was used to divide the 52 piglets from 17 L among the six experimental groups (n = 9): (1) handling: sham handling without injection and castration; (2) NaCl: injection with 0.9% sodium chloride using the one-step F method and castration without pain relief; (3) Lido1: 2% lidocaine hydrochloride using the one-step F method; (4) Lido2: 2% lidocaine hydrochloride using the two-step method; (5) Mepi1: 2% mepivacaine hydrochloride using the one-step F method; and (6) Mepi2: 2% mepivacaine hydrochloride using the two-step method (Table 2).

### 2.5. Anesthesia

Each piglet was anesthetized with the individually determined minimum alveolar concentration of isoflurane. Mask induction was performed with 5% isoflurane (Isoflurane Baxter vet., Baxter Deutschland GmbH, Unterschleißheim, Germany) in oxygen. The maintenance dose of isoflurane was individually evaluated for each piglet using an interdigital pinch to maintain light anesthesia. The depth of anesthesia was considered sufficient when animals still showed a pedal withdrawal reflex in response to an interdigital pinch, but the stimulus did not awaken the animals.

If no withdrawal reaction was observed, the isoflurane concentration was reduced by 0.2%. Prolonged paddling and/or movement of the forelimbs, back or head were judged as signs that the state of narcosis was too light, and the inspired isoflurane concentration was increased by 0.2%. After a three-minute stabilization period, another interdigital pinch was applied to the other hind limb and the concentration of inspiratory isoflurane was maintained or further increased depending on the movement intensity. After another stabilization period, the injection was performed.

The piglets breathed spontaneously during the entire period of narcosis. Inspiratory and expiratory isoflurane levels were continuously monitored (Vamos^®^ plus, Dräger Medical Deutschland GmbH, Unterhaching, Germany). A more detailed description of the experimental setup and the method used to adjust the depth of anesthesia may be found in Saller et al. [19]. After castration, anesthesia was maintained for another 90 min before the piglets were euthanized with an intravenous overdose of pentobarbital (Euthadorm 500 mg/mL Injektionslösung, CP Pharma, Hanover, Germany), and the spinal cord of each animal was then removed for Fos analysis.

### 2.6. Nociceptive Measurements

It was presumed that all piglets experienced similar stress from the basic experimental setup, such as induction of anesthesia and establishment of measurements.

For the application of the measurement devices and the final experimental procedure, piglets were placed in a supine position. Warm water bottles were arranged around the piglet to maintain body temperature and prevent cooling. Eye ointment (Bepanthen Augen- und Nasensalbe, Bayer Vital GmbH, Leverkusen, Germany) was applied, and cotton wool was placed in the external auditory canal to minimize the impact of background noise on the measurement results.

A local anesthetic cream (Emla^®^, AstraZeneca GmbH, Wedel, Germany) was applied to the ventral skin of the throat for 20 min before skin incision. The region for vascular access was infiltrated with a maximum of 0.3 mL lidocaine (2% lidocaine, bela-pharm Arzneimittelfabrik, Vechta, Germany) subcutaneously prior to the skin incision. With gentle preparation, a vascular access sheath (3F, Balt extrusion, Montmorency, France) was introduced into the left carotid artery, and invasive blood pressure was measured using a microtip catheter (FISO-LS Fiber Optic Pressure Catheter, FOP-LS-2FR-10, FISO Technologies Inc., Quebec, QC, Canada). Before each measurement, the catheter was calibrated to room air. Systolic blood pressure, diastolic blood pressure, MAP and an electrocardiogram (ECG) were recorded continuously (PLUGSYS module, EIM-B, EIM-A, heart rate module, HAEMODYN software, Hugo Sachs Elektronik—Harvard Apparatus GmbH, March-Hugstetten, Germany, FFP-LS and Evolution Software, FISO Technologies Inc., Quebec, QC, Canada). Baseline values for MAP and HR were determined over one minute prior to injection and castration. Mean MAP and HR values were calculated. Within one minute after each event, the maximum change from baseline was identified, and the percent deviation from baseline was determined.

During the entire period of anesthesia, oxygen saturation, HR (2500A VET, Nonin Medical Inc., Plymouth, MN, USA), body temperature (PLUGSYS Thermocouple Amplifier Module (TCAM), HAEMODYN software, Hugo Sachs Elektronik—Harvard Apparatus GmbH, March-Hugstetten, Germany), respiratory frequency and end-tidal CO_2_ were monitored (Vamos^®^plus, Dräger Medical Deutschland GmbH, Unterhaching, Germany).

Nocifensive movements were assessed in every piglet during injection, skin incision and cutting of the spermatic cord. The person who immobilized the piglet assessed the movement of the four limbs and the tension of the spine while blinded to the treatments. For the handling group, no blinding was possible because the differences were obvious (i.e., sham injection and sham castration).

The quality of the nocifensive movements was evaluated using a specific score adopted from Saller et al. [19]. All four limbs were assessed individually for each testicle as follows: score 1 = one movement, score 2 = two to three movements, and score 3 = more than three movements, long lasting. The back/spine was evaluated separately for each testicle: score 1 = muscle contraction and score 2 = movement. A maximum score of 28 points, with 14 points per testicle, could be reached per event (injection, skin incision and cutting of the spermatic cord).

### 2.7. Injection

The following local anesthetics were used: 2% lidocaine hydrochloride (Xylocitin^®^ 2%, Mibe GmbH Arzneimittel, Brehna, Germany) in the Lido1 and Lido2 groups and 2% mepivacaine hydrochloride (Mepidor^®^ 20 mg/mL solution for injection in horses, Richter Pharma AG, Wels, Austria) in the Mepi1 and Mepi2 groups. The NaCl group was injected with 0.9% sodium chloride (B. Braun, Melsungen, Germany) using the one-step F method. The injection was merely simulated in the handling group by fixing and gently touching the testes with a needle cap.

For both injection techniques, an automatic self–filling 1 mL syringe (HSW ECO–MATIC^®^, Henke-Sass, Wolf GmbH, Tuttlingen, Germany) was used, and needles were changed between each piglet. To standardize the procedure as much as possible, the same two persons always performed the injection and castration throughout the study. For the injection and castration procedures, one person constrained the piglets in the supine position with both palms under the back while keeping the hind limbs cranially using the thumbs. The person performing the injection and castration pressed the testicles caudally into the scrotal sac and fixed them between the thumb and index finger.

**Figure 1:**
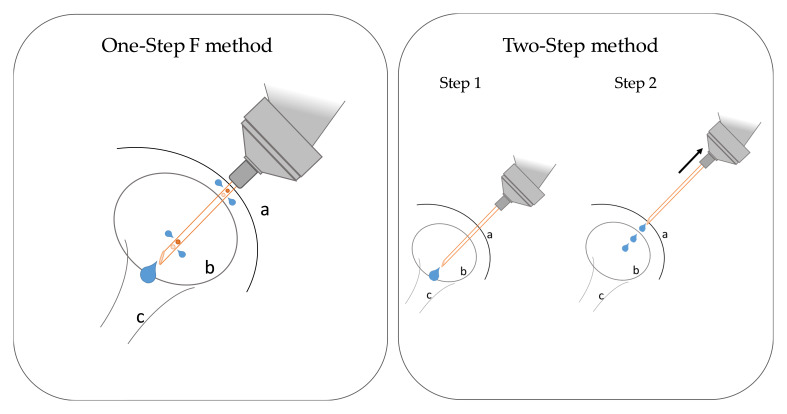
Schematic overview of the one-step F and two-step methods. a—Scrotal skin, b—testis, c—Spermatic cord. One step F method: the needle was inserted caudally to its full length so that the distal opening and the distal lateral opening were positioned in the testicle and the proximal lateral opening was arranged in the subcutaneous tissue of the scrotum. A total of 0.6 mL of a local anesthetic was administered. Two-step method: Step (1) the needle was inserted for approximately three-fourths of its length and a volume of 0.4 mL of the local anesthetic was intratesticularly injected. Step (2) The needle was withdrawn, and a volume of 0.2 mL was evenly distributed over the injection canal.

The one-step F injection was performed using a special cannula invented by the authors. The objective in developing this device was to create a cannula for administering a local anesthetic for castration of a piglet that is easy to handle and facilitates efficient and proper local anesthetization of the testicles and scrotal skin. The newly invented 25 G cannula (0.5 × 10 mm) contained the usual distal opening and two proximal and two distal lateral openings for simultaneous release of the local anesthetic. The specialized cannulas were manufactured individually. The lateral holes were drilled using laser technology. The needle was inserted caudally to its full length so that the distal opening and the distal lateral opening were positioned in the testicle and the proximal lateral opening was arranged in the subcutaneous tissue of the scrotum (Figure 1). A volume of 0.6 mL of a local anesthetic (Lido1, Mepi1) or sodium chloride (NaCl) was administered per side.

The two-step injection was modified by Hansson et al. [11]. In the first step, a commercially available 25 G needle (0.5 × 16 mm, B. Braun TravaCare GmbH, Hallbergmoos, Germany) was inserted caudally for approximately three-fourths of its length so that the tip of the needle was positioned intratesticularly. A volume of 0.4 mL of the local anesthetic was carefully injected. In the second step, the needle was withdrawn, and a volume of approximately 0.2 mL was evenly distributed over the injection canal to the surface (Figure 1). In total, approximately 0.6 mL was injected per testis.

### 2.8. Castration

Surgical castration was performed 20 min after injection to ensure that both local anesthetics were fully effective. Two vertical skin incisions were made parallel to the raphe scroti using a scalpel and the testicles were gently pressed out. To evaluate noxious stimulation of the skin incision independently, a period of two minutes was allowed to pass for stabilization of the parameters before the spermatic cords were severed using an emasculator. Castration was merely simulated in the handling group by holding the intact testicles in position and gently touching the skin with the blunt back of the scalpel handle.

### 2.9. Fos Protein Expression

Ninety minutes after the castration was finished, the piglet was euthanized, and lumbar and sacral segments of the spinal cord were carefully removed from the vertebral canal. Using the vertebrae as landmarks, the spinal cord was divided into lumbar (L1, L2, L3) and sacral (S1–S3) spinal cord segments, fixed in 38% (*w*/*w*) neutral buffered formaldehyde for at least 48 h and embedded in paraffin (Leica ASP300S, Leica, Wetzlar, Germany). Cross sections (2 µm) were produced starting from the cranial cut surface. Serial sections were stained with hematoxylin and eosin using a standard protocol. Immunohistochemistry was performed to investigate Fos protein expression (anti-c-Fos antibody, ab209794, Abcam, Cambridge, UK, diluted in antibody diluent 1:100) using a Leica Bond RXm system. Briefly, after deparaffinization, epitope retrieval 1 was performed using citrate buffer (pH 6) for 30 min. For primary antibody binding detection and visualization, a Polymer Refine Detection kit with 3,3’-diaminobenzidine (DAB) and no secondary antibody was used. Slides were digitized using a Leica AT2 scanning system. The spinal dorsal horn was identified as the gray matter dorsal to a line drawn from the central canal to the lateral border of the gray matter using Aperio Imagescope Software version 12.4.0.7081 (Leica Biosystems, Wetzlar, Germany). A German board-certified pathologist blinded to the sample identities performed semiquantitative analysis of the staining intensity of Fos-positive neurons. Four different segments of each animal were evaluated. Scores were assigned as follows: 0 = no staining, 1 = slight staining in single neurons (≤25%), 2 = slight staining in some neurons (≤50%), 3 = moderate staining in some neurons (≤50%), 4 = strong staining in some neurons (≤50%), 5 = strong staining in all neurons.

### 2.10. Data and Statistical Analysis

For the statistical analysis, groups were tested for changes in MAP, HR and Fos protein expression, and significant differences in age, weight and end-tidal isoflurane concentration and nocifensive movements were observed.

The distribution of all continuous parameters was tested using the Shapiro–Wilk normality test. One-way ANOVA was performed for data with normally distributed residuals. If significant, pairwise Student’s *t*-tests or Games–Howell tests with Benjamini and Hochberg *p*-value adjustment for multiple comparisons were performed. The Kruskal–Wallis test was performed for non-normally distributed residuals. If significant, Dunn’s test of multiple comparisons using rank sums with Benjamini and Hochberg *p*-value adjustment for multiple comparisons was performed.

For the statistical analysis of the distribution, an independent sample *t*-test was used. For the distribution score, the intraclass correlation coefficients (ICCs) and their 95% confidence intervals were calculated using IBM SPSS Statistics for Mac version 1.0.0.1508. Based on the mean rating, absolute agreement and 2-way mixed-effects model, the ICC values ranged between 0 and 1, with values closer to 1 indicating stronger reliability.

Statistical significance was considered at *p* ≤ 0.05. Statistical analyses were performed using R version 3.6.1 (2019-07-05) and GraphPad Prism 9.0.2.

## 3. Results

### 3.1. Distribution

**Figure 2:**
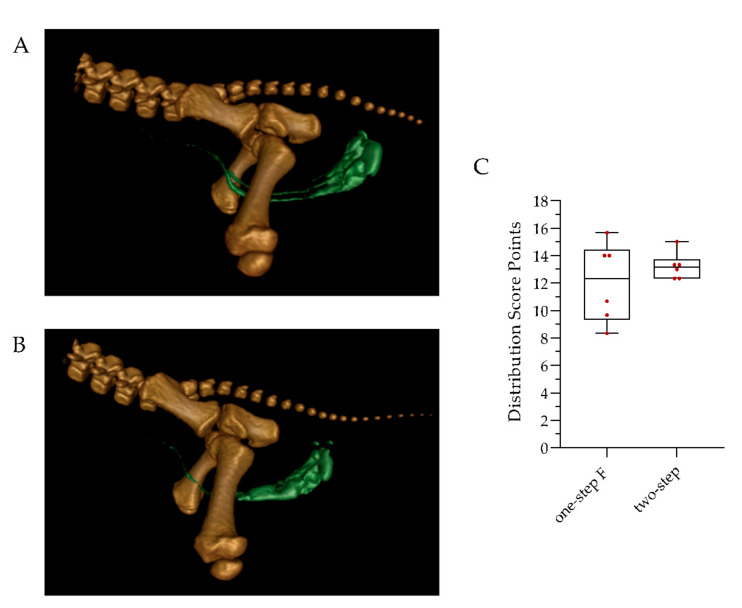
Distribution of the lidocaine/contrast medium mixture directly after injection, using the one-step F and two-step methods. Exemplary three-dimensional reconstruction of a piglet injected with one step F (**A**) and a piglet injected using the two-step method (**B**). Bone structures such as parts of the spine, pelvis and femur are colored brown. The injected lidocaine/contrast agent mixture is colored green. (**C**) CT distribution scoring in piglets injected with the one-step F or two-step method.

**Figure A1:**
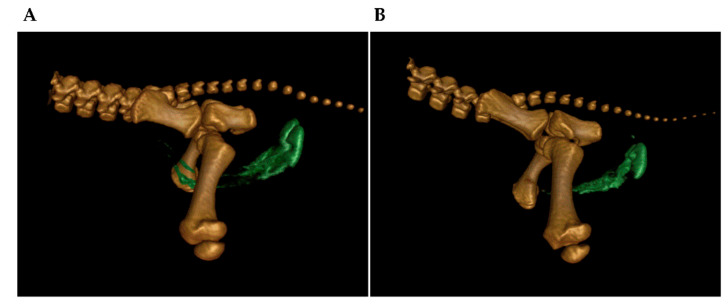
Distribution of the lidocaine/contrast medium mixture 20 min after injection using the one-step F and two-step methods. Exemplary three-dimensional reconstruction of a piglet injected using the one-step F (**A**) and a piglet injected using the two-step method (**B**). Bone structures such as parts of the spine, pelvis and femur are colored brown. The injected lidocaine/contrast agent mixture is colored green.

The distribution of a contrast agent diluted in 2% lidocaine hydrochloride injected via two different application methods was visualized using CT (Figure 2A,B). The one-step F and two-step injection achieved a widespread distribution of the lidocaine/contrast agent mixture in the scrotal skin, testicles, testicular sheaths and spermatic cord directly after administration. No further spreading of the contrast agent was observed during the 20 min after injection (Figure A1). The distribution and accumulation of the contrast medium were evaluated based on a CT distribution score (Table 1). The ICC for the evaluation of the distribution of the contrast agents was 0.89 (confidence interval (CI): 0.76–0.96). No significant difference was observed between the one-step F and two-step injection groups (Figure 2C). Both injection techniques produced similar distribution patterns and reached comparable distribution scores.

### 3.2. Injection Pressure and Injection Time

**Figure 3:**
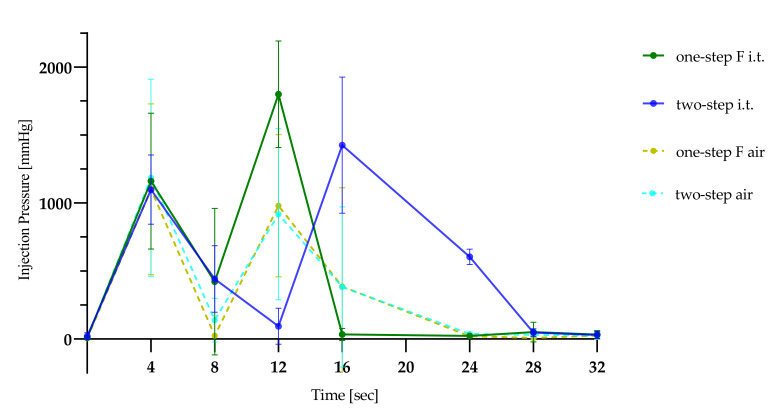
Injection pressure profiles of the two injection methods into the testis and air. i.t. = intratesticular.

Figure 3 shows the injection pressures during injections into the air and testicular tissue using the one-step F and two-step injection methods. A 0.6 mL intratesticular injection of 0.9% sodium chloride using the one-step F method generated a maximum pressure of 1572.67 ± 381.53 mmHg. The injection of 0.4 mL intratesticularly and 0.2 mL subcutaneously using the two-step method created a maximum pressure of 1486.64 ± 461.46 mmHg. One-step F injection exceeded the maximal measurable pressure in four of seven animals, and it was observed in one of four animals using the two-step method. Measuring the injection pressure in air resulted in an average pressure of 1215.87 ± 308.11 mmHg with the one-step F method and 1083.33 ± 714.24 mmHg with the two-step injection method. The mean execution time for the one-step F injection, including both testicles, was 14 ± 1.6 s. The two-step method was more time consuming, with a mean of 23 ± 2.3 s.

### 3.3. Group Homogeneity

Two piglets from the Lido1 group were excluded due to incomplete data acquisition. The treatment groups were homogeneously distributed in age, weight and end-tidal isoflurane concentration during injection and castration (Table 3) and did not significantly differ between groups. The mean end-tidal isoflurane of all piglets in this study was 1.4 ± 0.35% with a flow of 3 L min^−1^ oxygen. The local anesthetic dosage was the same for each piglet, regardless of the individual piglet weight. Therefore, the administered local anesthetic dose per weight varied between the animals. The highest lidocaine dose (19.29 mg/kg) was administered in the Lido2 group, and the lowest dose was administered in the Lido1 group (7.74 mg/kg). For mepivacaine, the highest dosage per weight was given in the Mepi1 group (17.14 mg/kg), and the lowest dosage (7.87 mg/kg) was given in the Mepi2 group. None of the piglets injected with a local anesthetic showed signs of incompatibility or side effects.

### 3.4. Blood Pressure and Heart Rate

#### 3.4.1. Interdigital Pinch

**Figure A2:**
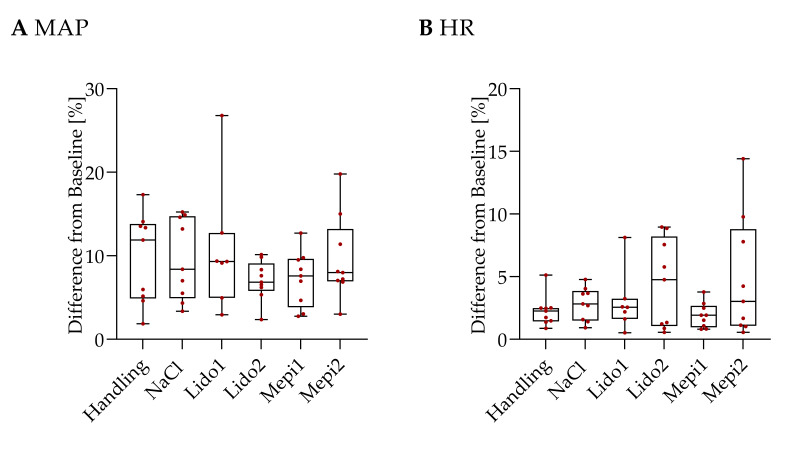
Interdigital pinch. Percent change in (**A**) mean arterial blood pressure (MAP) and (**B**) heart rate (HR) during the interdigital pinch for the adjustment of anesthesia depth. Values shown are means ± SD.

The measured baseline mean MAP for all piglets was 49.94 ± 6.04 mmHg. The depth of anesthesia was individually adjusted using an interdigital pinch. The observed changes in blood pressure as a nociceptive reaction to the interdigital pinch were similar between groups, and no significant differences were observed (Figure A2). The same results were obtained for changes in heart rate during interdigital pinch.

#### 3.4.2. Injection

**Figure 4:**
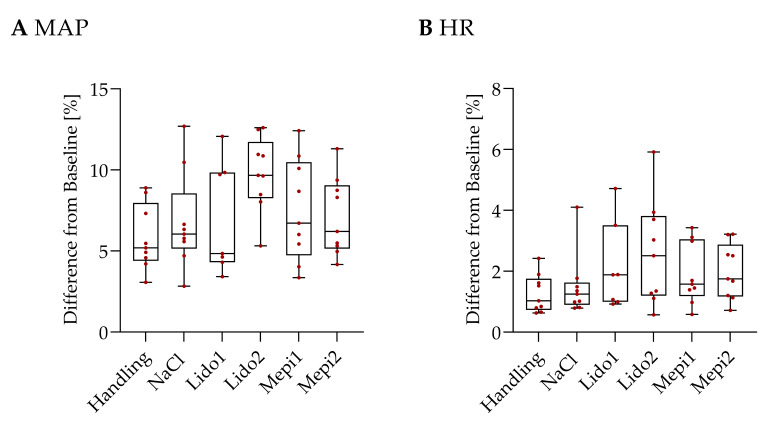
Injection. Percent change in MAP (**A**) and HR (**B**) during injection. Values shown are means ± SD.

Injections of a local anesthetic or sodium chloride provoked no significant changes in MAP or HR compared to that of the handling group, in which the injection was only simulated (Figure 4).

#### 3.4.3. Castration

**Figure 5:**
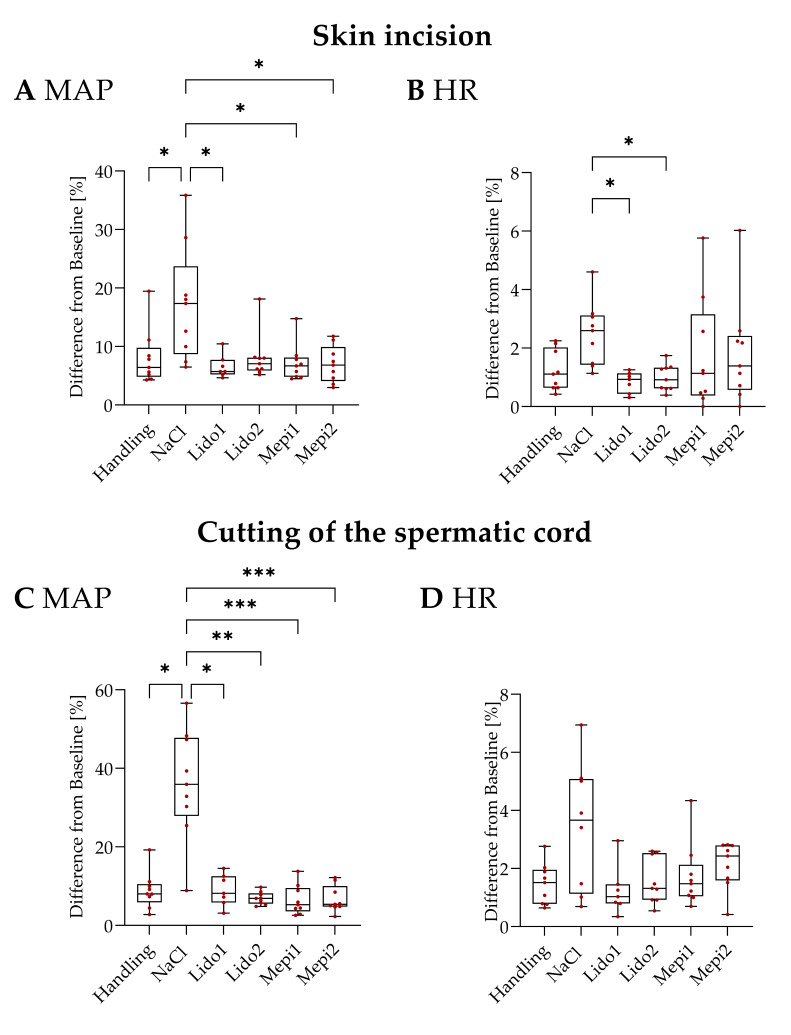
Castration. Percent change in MAP (**A**,**C**) and HR (**B**,**D**) during skin incision (**A**,**B**) and cutting of the spermatic cord (**C**,**D**). Values shown are means ± SD. Statistical significance is indicated as * *p* ≤ 0.05, ** *p* < 0.01, *** *p* < 0.001.

Figure 5A shows the MAP changes during skin incision. Cutting of the scrotal skin without pain relief (NaCl) provoked a 17.24% maximal change in MAP from baseline. In contrast, the handling group showed a significantly lower MAP (*p* = 0.037). Administration of a local anesthetic, except for Lido2, caused significantly reduced changes in MAP compared to NaCl (Lido1: *p* = 0.034, Mepi1: *p* = 0.034, Mepi2: *p* = 0.034) and was comparable to the handling group. Lido1 (*p* = 0.038) and Lido2 (*p* = 0.044) achieved a significant reduction in HR changes compared to NaCl (Figure 5B). Among the other groups, no significant differences in HR were observed during the skin incision.

During cutting of the spermatic cord, the NaCl group responded with the highest changes in MAP (36.10%) and was significantly increased compared to the handling piglets (*p* = 0.016) (Figure 5C). Compared to NaCl, the MAP of the four local anesthetic groups (Lido1: *p* = 0.044, Lido2: *p* = 0.002, Mepi1: *p* = 0.001, and Mepi2: *p* = 0.001) was significantly decreased. The MAP of the groups receiving a local anesthetic was comparable to the handling group. No significant differences in HR deviation were detected between the treatment and control groups after cutting the spermatic cord (Figure 5D).

### 3.5. Nocifensive Movements

As shown in Table 4, only Mepi1 showed significantly more nocifensive movements during injection than Lido2 (*p* = 0.031) and Handling (*p* = 0.031). The highest proportion of piglets showing no signs of nocifensive movements according to injection were observed in the Handling and Lido2 groups. No significant differences were observed during skin incisions between the groups. The NaCl group had the highest number of piglets responding to skin incisions and achieved the highest score (5.1 ± 6.7) among the other groups. Handling and Lido1 piglets did not move at all during the skin incision. For cutting the spermatic cord, NaCl produced the highest mean score (10.9 ± 8.7), and most piglets (eight of nine) showed nocifensive movements in this group. Significantly lower nocifensive scores (*p* ≤ 0.001) were found for all experimental groups and the handling group. Handling and Mepi2 piglets showed no nocifensive movements, but only one piglet in each of the other local anesthesia groups reacted to cutting of the spermatic cord.

### 3.6. Fos Protein Expression

**Figure 6:**
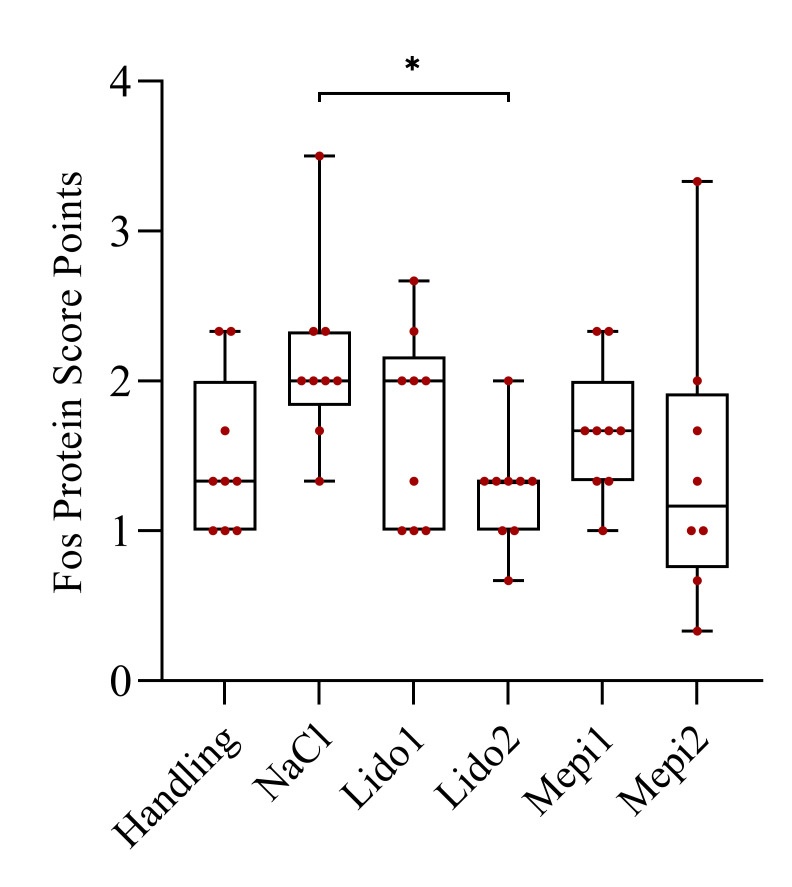
Fos protein expression in the lumbar and sacral segments of the spinal cord 90 min after castration. * *p* ≤ 0.05.

Figure 6 summarizes the Fos protein expression in the lumbar and sacral segments of the spinal cords of the six treatment groups using a semiquantitative score based on the staining intensity. Significantly reduced Fos protein expression compared to the NaCl control was found only in Lido2 (*p* = 0.016).

## 4. Discussion

Local anesthesia for piglet castration has been investigated in many studies, but divergent outcomes have been reported. It is difficult to compare the results of these investigations due to their very different study designs. Different local anesthetics, evaluation criteria, injection methods and volumes were used. Most studies used an intratesticular injection [1,2,15,21,22,23,24,25,26,27,28,29], or the injection into the testis was combined with a subcutaneous depot [1,10,11,12,14,17,18,19,20,26,30,31,32,33]. Other studies [2,10,14,17,18] chose an intrafunicular and subcutaneous approach, and Zankl et al. and White et al. injected local anesthetics only intrascrotally [15,34]. Sutherland et al. examined a needle-free injection through the skin [35]. Notably, the exact injection procedure is not described in detail in many publications, which may explain the poor reproducibility of some studies. The present study investigated two different injection methods: the two-step method, modified according to Hansson et al. [11], and the one-step F method, which used a newly developed cannula with additional lateral openings. The focus of both methods was achieving practicability for farmers in practice while maintaining high efficacy.

The complex innervation of the testicular region and the variety of anatomical structures that must undergo sensory block for painless castration are challenging in the administration of local anesthesia. Sources of porcine testis innervation are pelvic, sensory, pre- and paravertebral ganglia. The main nerve supply is provided by the anterior pelvic ganglion and the caudal mesenteric ganglion [36]. Originating from the caudal mesenteric ganglion, the testicular plexus runs together with the testicular artery and innervates the testis and epididymis [37]. The innervation of the scrotum originates from the nervi scrotales dorsales, which are end branches of the pudendal nerve, and the tunica vaginalis and the cremaster muscle are supplied by branches of the genitofemoral nerve [37].

CT imaging showed that the one-step F and two-step injection methods resulted in an even distribution within the scrotal skin, testicular sheath, testis and spermatic cord directly after injection. Although only the distribution of the contrast agent can be followed with CT, we presumed that the local anesthetic was distributed in a similar manner. This presumption is supported by Ranheim et al. [33], where intratesticularly applied radiolabeled lidocaine was rapidly transported into the spermatic cord and maximum enrichment was measured after three minutes. For better comparability, a 20 min waiting time before castration was used, based on our previous studies [19,20]. Faster times of onset of several minutes were reported for lidocaine and mepivacaine [38], which suggests that a shorter waiting period to perform castration may be sufficient. A shortening of the waiting period has been applied in other studies and may lead to more flexibility in the implementation of the method in practice [14].

The tunica albuginea is a fibrous tissue capsule that covers the testis and prevents the testicle from expanding [39]. This rough capsule limits intratesticularly injected fluids, and the resulting high pressure in the testicle pushes the fluid toward the spermatic cord [33]. The injection pressures achieved with both methods into the testis are comparable to intraneural or intradermal injections [40,41]. However, these data should be interpreted with caution because the measurement system only detected pressures up to 2121 mmHg. The pressure limit was reached five times, four times in the one-step F group and once in the two-step group. Therefore, we do not know exactly the intensity of the pressure. The injections into the air reached similar values, which indicates that the resistance within the syringe system and the cannula was already high. The small diameters of the cannulas and the injection speed may lead to this high base pressure. Injection speed (mL per second) was not supervised in this study, but the same two persons performed injections to standardize the injection as much as possible. Other factors, such as needle diameter and length, injection volume, temperature of the fluid and drug formulation, influence the perception of pain during injections [42]. The injection of local anesthetics provoked nocifensive movements in awake piglets [20]. Therefore, we further tried to minimize injection pain by reducing the total injection volume from 1 mL to 0.6 mL per side. Although only Mepi1 reached significantly higher nocifensive movement scores than Lido2 and Handling in our study, many piglets in the NaCl, Mepi2 and Lido1 groups showed nocifensive reactions. However, these reactions were not significant. To improve the observed reactions, the total volume may be further reduced. However, a sufficient analgesic effect must be ensured. Previous studies did not fully prevent the discomfort of intratesticular injections by volume reduction [10]. Further efforts to minimize injection pain may include the use of thinner needles or to buffer local anesthetics [43]. Buffering often automatically increases the injection volume when there are no more concentrated products on the market. The addition of bicarbonate is a medicinal product preparation that may not be performed by the farmer. Whether an injection into the testis may occur without any sensation of pain and whether the injection should be included in the evaluation of local anesthesia as a legal alternative for the castration of conscious piglets must be discussed.

For the time requirement, a comparison of both techniques revealed that the one-step injection took much less time than the two-step injection. The impression of the veterinarian operators was that the practical implementation of the two-step injection was more challenging than that of the one-step F. The one-step F injection required only one simple placement of the cannula into the testis. Due to the additional lateral holes, no repositioning of the cannula for subcutaneous infiltration was necessary. One issue that occurred during the performance of the second step of the two-step method was the difficulty of injecting an exact volume of only 0.2 mL while withdrawing the cannula. This difficulty occurred because an automatic self-filling syringe with a preset application volume of 0.4 mL was used for the first step. A second volume could not be preset, which made the exact dosing for the second step more difficult. Skade et al. [10] compared two injection methods and favored a faster and less complicated method for practical application. As a next step, the injection methods must be evaluated in the field of awake animals to draw a final conclusion.

Local anesthetic solutions do not contain epinephrine because vasoconstrictors could affect blood pressure and heart rate measurements [19]. Both injection techniques and both local anesthetics achieved a significant reduction in nocifensive movements and MAP during cutting of the spermatic cord, which is the most painful part of castration [44]. Only the combination of lidocaine and the two-step method did not achieve a significant reduction in MAP during skin incisions. For nocifensive movements during skin incision, there was no significant difference between piglets castrated without pain relief and piglets that received a local anesthetic. The fact that not every piglet from the NaCl group showed nocifensive movements could be because the piglets were under light isoflurane anesthesia. Although isoflurane was individually titrated for each piglet to reach a state of hypnosis, as indicated by the presence of a pedal withdrawal reflex, anesthesia could mask possible movements. The reason why individual piglets of the local anesthesia groups still moved during skin incision and cutting of the spermatic cord, despite local anesthesia, may be that nociception was not fully blocked in some piglets. Another explanation may be that external influences, such as fixation of the testes, affected the piglet’s reactions because one animal in the handling group also showed defensive reactions during simulated injection.

Fos protein is expressed in neurons of the dorsal horn of the spinal cord after nociceptive stimulation and it is used as a marker for pain [45]. Fos protein expression reflects only the sum of the pain experienced and cannot be assigned to one specific pain event. Nyborg et al. [46] showed that injection of a local anesthetic before castration reduced the number of Fos-positive neurons in the dorsal horn. Reiser et al. found a significant reduction in the Fos staining score in piglets castrated after an injection of lidocaine, mepivacaine and procaine [47]. Only Mepi1 achieved a significant reduction in Fos protein expression compared to the NaCl castrated piglets in the present study. Piglets that did not experience injection or castration also expressed the Fos protein. This expression may be explained by the fact that stress or unpleasant stimulation, such as fixation of the piglet itself and especially the testicles, also increases Fos protein expression in the dorsal horn. The neonatal age of the piglets may explain this result. For example, a Fos response to innocuous stimulation was observed in neonatal rats until postnatal day 21 [48]. Therefore, Fos protein expression should not be used as the sole parameter to assess nociception in piglets following castration, and it should always be evaluated in combination with other parameters.

The sample size for this study was calculated based on MAP, which is the most sensitive cardiovascular parameter associated with noxious stimuli during castration [19,49]. Therefore, the power of the study may be too low to draw definite conclusions on nocifensive movements and Fos expression, and a direct comparison of the injection methods and local anesthetics is difficult. The present study only evaluated nociceptive reactions in response to acute stimuli under light isoflurane anesthesia. Chronic effects and practicability in awake piglets must be evaluated in further ongoing studies.

## 5. Conclusions

In conclusion, both injection techniques, regardless of the local anesthetic used, reduced nociception during castration of male piglets under standardized conditions. One challenge remains the handling of the discomfort of the injection itself, which continued to elicit nocifensive movements in several piglets from the different experimental groups. For speed and manageability, the one-step F method was beneficial. However, compared to the two-step method, the applied pressure was higher and more frequently exceeded the maximal measurable pressure. Both injection techniques must be further evaluated in conscious piglets to draw final conclusions about their effectiveness and feasibility in practical use.

## Figures and Tables

**Table 1:** CT distribution scoring system.

Anatomical Structures	Distribution	Accumulation	Score
Scrotal skin	n.a.	no	0
	n.a.	yes	1
Testis	−	−	0
	+	+	1
	+	++	2
	++	+	
	++	++	3
Testicular sheath	−	−	0
	+	+	1
	+	++	2
	++	+	
	++	++	3
Spermatic cord	−	−	0
	+	+	1
	++	++	2
Maximum score per testis			9
Maximum score per piglet			18

n.a. = not assessed, − = not present, + = moderate, ++ = intense.

**Table 2:** Classification of study groups.

	Handling	NaCl	Lido1	Lido2	Mepi1	Mepi2
n per group	9	9	7	9	9	9
Injection method	Sham	One-step F	One-step F	Two-step	One-step F	Two-step
Injection solution	-	NaCl	Lidocaine 2%	Lidocaine 2%	Mepivacaine 2%	Mepivacaine 2%
Injection volume	-	0.6 mL	0.6 mL	0.4 + 0.2 mL	0.6 mL	0.4 + 0.2 mL
Castration	Sham	Yes	Yes	Yes	Yes	Yes

Sham injection: The piglet was fixed for injection. The testicles were fixed and merely touched with a needle cap. Sham castration: The piglet was fixed for castration. The testicles were fixed, and a skin incision was simulated with the back of the scalpel handle.

**Table 3:** Mean values and standard deviation of age, body weight, local anesthetic dosing, and end-tidal isoflurane concentration during injection and castration.

	Handling	NaCl	Lido1	Lido2	Mepi1	Mepi2
Age (days)	4.78 ± 0.79	5.44 ± 1.17	4.57 ± 1.36	4.22 ± 1.13	5 ± 1.33	4.89 ± 1.45
Weight (kg)	2.01 ± 0.51	2.32 ± 0.69	2.37 ± 0.41	2.04 ± 0.43	1.98 ± 0.38	2.21 ± 0.50
Local anesthetic dosing (mg/kg) *			10.4 ± 1.65	13.8 ± 3.07	12.01 ± 2.80	12.61 ± 2.5
End-tidal Isoflurane–injection (Vol %)	1.46 ± 0.38	1.32 ± 0.26	1.4 ± 0.20	1.39 ± 0.44	1.38 ± 0.18	1.57 ± 0.38
End-tidal Isoflurane–castration (Vol %)	1.52 ± 0.48	1.31 ± 0.26	1.46 ± 0.22	1.38 ± 0.44	1.39 ± 0.16	1.62 ± 0.38

* The values refer only to the amounts applied to the testicular region and do not include infiltration of the neck with approximately 0.3 mL of 2% lidocaine for the catheter implementation.

**Table 4:** Nocifensive scores mean ± SD and number of piglets without nocifensive movements during injection, skin incision and cutting of the spermatic cord.

		Handling	NaCl	Lido1	Lido2	Mepi1	Mepi2
**Injection**							
Score	mean	0.1 ^a^	0.8	1.3	0.8 ^a^	7.1 ^b^	2.7
SD		0.3	1.6	2.1	2.2	6.5	3.2
Animals without nocifensive movements	n	8/9	5/9	4/7	8/9	3/9	4/9
**Skin incision**							
Score	mean	0	5.1	0	1.7	3.1	2.1
SD		0	6.7	0	3.1	8.8	4.0
Animals without nocifensive movements	n	9/9	5/9	7/7	6/9	8/9	6/9
**Cutting of the spermatic cord**							
Score	mean	0 ^a^	10.9 ^b^	0.9 ^a^	0.1 ^a^	0.7 ^a^	0 ^a^
SD		0	8.7	2.1	0.3	1.9	0
Animals without nocifensive movements	n	9/9	1/9	6/7	8/9	8/9	9/9

^a,b^ Differing superscripts within one line indicate significant (*p* < 0.05) differences between groups.

## Data Availability

Not applicable.
